# Neuro-Oncology Biotech Industry Progress Report 2025

**DOI:** 10.7759/cureus.106278

**Published:** 2026-04-01

**Authors:** Zoey A Croft, Griffin Thomas, Evan Keister, Maille McDermott, Shoaib Syed, Samuel Latzman, Tamika Wong, Yafell Serulle, David J Langer, Randy D'Amico, John Boockvar

**Affiliations:** 1 Neurosurgery, Lenox Hill Hospital, New York, USA; 2 Neurosurgery, Lenox Hill Hospital/Donald and Barbara Zucker School of Medicine at Hofstra/Northwell, New York, USA

**Keywords:** biotechnology, brain tumor, glioblastoma, glioma, neuro-oncology

## Abstract

Neuro-oncology is central to the rapidly expanding field of cancer therapeutics, with surges in clinical trials exploring innovative treatment strategies. The 2025 Brain Tumor Biotech Summit was hosted by Northwell Health Lenox Hill Hospital’s Department of Neurosurgery, the Feinstein Institute for Medical Research, and the Zucker School of Medicine in New York City in June 2025. The summit highlighted recent advancements in neuro-oncology, with a focus on emerging treatments, novel drug delivery platforms, and device-based therapeutics. Leaders from academia, biotechnology, and investment communities presented progress on clinical trials, early-phase technologies, and personalized treatment strategies for glioblastoma (GBM) and other CNS tumors. This report summarizes the key discussions at the summit and emphasizes the importance of translational partnerships in accelerating therapy development for glioma patients.

## Introduction and background

Despite decades of intensive research and numerous clinical trials, prognoses for patients with malignant brain tumors, particularly glioblastoma, remain dismal. Standard-of-care therapy, including surgical resection followed by radiation, has only modestly improved with the addition of temozolomide (TMZ) chemotherapy, extending typical survival from 12 to 14 months. The heterogeneous nature of gliomas, combined with the challenge of drug delivery across the blood-brain barrier (BBB), continues to test the boundaries of conventional treatment [1, 2].

Traditional federal funding streams for large-scale clinical trials and translational research remain limited and vulnerable to budgetary constraints. In response, collaborations with biotechnology companies, private investors, non-profit organizations, and philanthropic initiatives have aimed to fill the funding gap and accelerate the path of translational research from the bench to the bedside. This intersection of science, industry, and investment has become a defining feature of transformative advances in neuro-oncology.

Historically, the Brain Tumor Biotech Summit has served as a forum for researchers and companies to discuss the latest innovations in neuro-oncology biannually in New York City. Table 1 and Table 2 provide concise lists of product and financial updates.

**Table 1:** Summary of companies and therapies/products with current status. TTFields, Tumor Treating Fields; FDA, Food and Drug Administration; SDT, sonodynamic therapy; 5-ALA, 5-aminolevulinic acid; HGG, high-grade glioma; DIPG, diffuse intrinsic pontine glioma; IGV, ImmunoGene vaccine; CNS, central nervous system; TMZ, temozolomide; Y90, yttrium-90; 3D, three-dimensional; PET-CT, positron emission tomography–computed tomography; GBM, glioblastoma multiforme; FTB, fractional tumor burden.

Company	Product/platform	Mechanism/purpose	Trial status
Novocure (Baar, Switzerland)	Optune/TTFields	Tumor cell division disruption via electric fields	FDA approved
Alpheus Medical (Chanhassen, MN, USA)	SDT + 5-ALA	Ultrasound-activated 5-ALA induces selective tumor cell death in recurrent HGG	Phase I/II
SonALAsense (Oakland, CA, USA)	SDT + 5-ALA	Ultrasound-activated 5-ALA induces selective tumor cell death in DIPG	Phase I/II
Imvax (Philadelphia, PA, USA)	IGV-001	Autologous immunotherapy using antisense-treated tumor cells in diffusion chamber	Phase II
Harvard Mass Gen (Boston, MA, USA)	CARV3-TEAM-ET	Intraventricular CAR-T targeting EGFRvIII & IL13Rα2	Preclinical
CNS Pharmaceuticals (Houston, TX, USA)	TPI 287	Brain-penetrant taxane stabilizing microtubules, overcomes TMZ resistance	Phase I
Boston Scientific (Marlborough, MA, USA)	Intra-arterial Y90	Selective radioembolization using Y90-loaded microspheres	Phase I
DNKO (https://www.dnko.net)	Thermotherapy + liposomes	Heat-activated liposomes release chemo agents directly in tumor tissue	Preclinical
Kiyatec (Greenville, SC, USA)	3D cell culture	Patient-derived 3D tumor models to predict chemotherapy response	Clinical validation
Oncovision (Boston, MA, USA)	CareMiBrain PET-CT	Dedicated PET-CT for targeted imaging of GBM metabolism and planning	Clinical use
Imaging Biometrics (Elm Grove, WI, USA)	FTB MRI mapping	MRI-based biomarker distinguishes tumor progression from pseudoprogression	Clinical/FDA cleared
Houston Methodist (Houston, TX, USA)	Oncomagnetic device therapy	Oscillating magnetic wave therapy for treatment-resistant brain tumors	FDA expanded access

**Table 2:** Recent financial updates from presenting companies. FDA, Food and Drug Administration; TTFields, Tumor Treating Fields; NIH, National Institutes of Health; SBIR, Small Business Innovation Research.

Company	News
Imvax (Philadelphia, PA, USA)	Raised $112M in Series C funding led by Blackstone Life Sciences and Ziff Capital Partners (2023–24)
Novocure (Baar, Switzerland)	$100M investment from Pharmakon Advisors (2023); revenue expansion following FDA approvals for TTFields
DNKO (https://www.dnko.net)	Early-stage biotech; pre-seed fundraising in progress
Kiyatec (Greenville, SC, USA)	Received NIH SBIR grant; expanded commercial contracts with academic cancer centers (2023–24)
CereVasc (Boston, MA, USA)	Raised over $70M in venture capital; pilot trials funded through Breakthrough Device support

**Figure 1:**
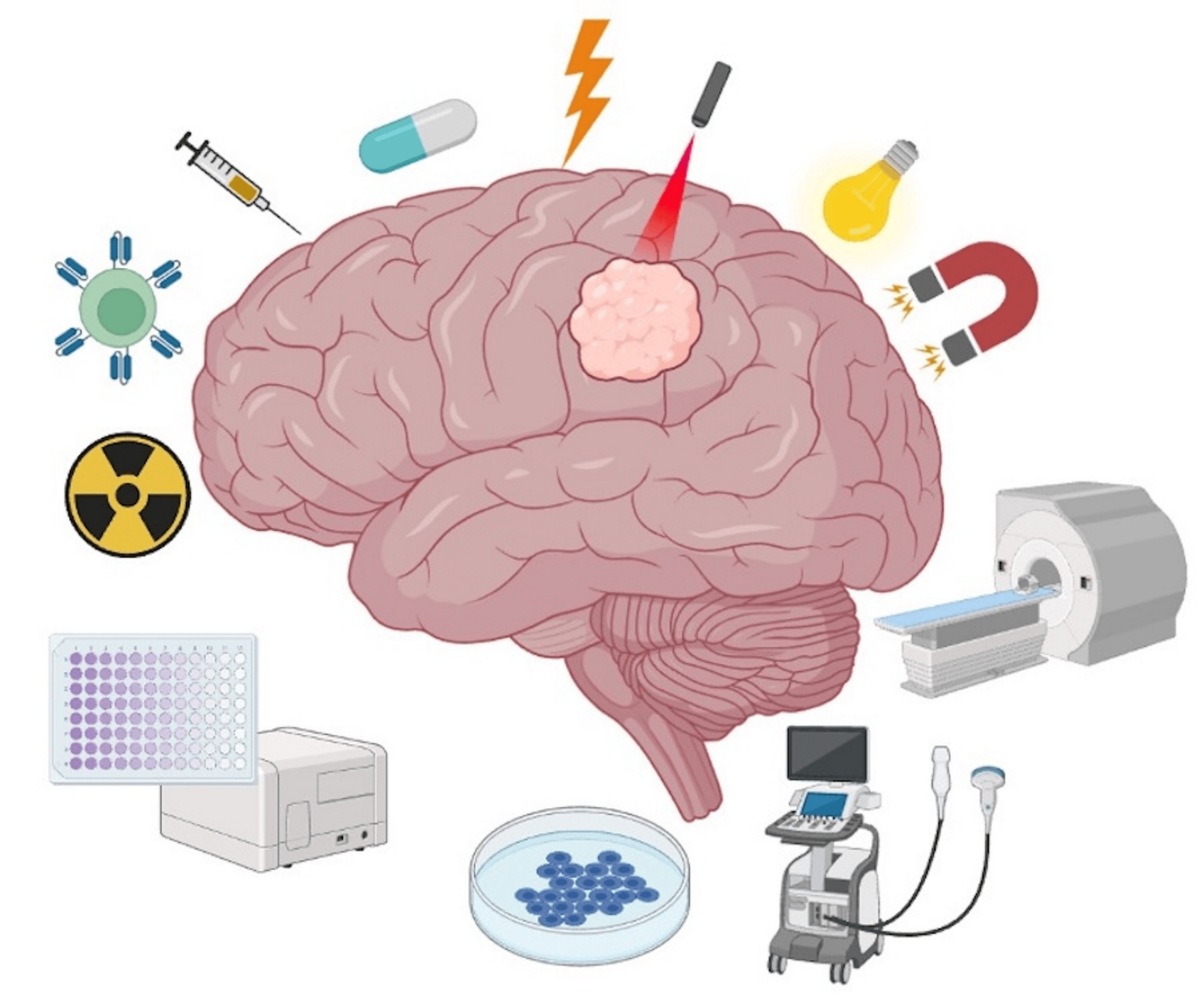
Multimodal approaches in brain tumor therapy. Schematic representation of emerging strategies in neuro-oncology illustrating the integration of surgical, pharmacologic, and device-based modalities, including chemotherapeutics, immunotherapy, radiation, phototherapy, focused ultrasound, and advanced imaging, among others. Created in BioRender. Keister, E. (2025) https://biorender.com/a642ogd

Figure 1 offers a visual representation of the therapeutic strategies discussed. This report summarizes the key discussions at the summit and emphasizes the importance of translational partnerships in accelerating therapy development for glioma patients. All the information in the following sections is publicly available for further reference.

## Review

Novel treatment products

Tumor Antigen Targeting Therapy

Imvax, Inc. (Philadelphia, PA, USA) uses its proprietary Goldspire® platform to generate IGV-001, which combines autologous GBM cells harvested during tumor resection with IMV-001, an antisense oligonucleotide that targets the coding region of the insulin-like growth factor type 1 receptor (IGF-1R) [3, 4]. The cells plus additional IMV-001 are combined in a biodiffusion chamber, irradiated, and implanted in the patient’s abdominal wall approximately 48 hours after tumor resection [3]. Explantation occurs 48 hours afterwards. IGV-001 primes anti-tumor immunity by presenting a broad tumor antigenic payload as cells undergo apoptosis.

In a Phase Ib trial, the addition of Imvax to standard of care (SOC) radiation and TMZ extended progression-free survival (PFS) to 9.8 months, compared with 6.5 months with SOC alone [4]. A multicenter, randomized, double-blind Phase IIb trial is currently underway to further evaluate efficacy [4].

Immunotherapy

Use of chimeric antigen receptor (CAR) T-cell therapy in the treatment of solid tumors, such as glioblastoma (GBM), has been limited by its inability to target the heterogeneous surface antigens across tumor cells. The investigational CAR T-cell therapy, CARv3-TEAM-E, addresses antigenic heterogeneity in recurrent GBM. The construct combines a CAR targeting the tumor-specific EGFRvIII mutation, which portends a poor prognosis, together with a secreted T-cell-engaging antibody molecule (TEAM) that binds wild-type EGFR, enabling recognition of both mutant and wild-type receptor expression in glioma cells [5].

In a Phase I first-in-human study, a single intraventricular administration of CARv3-TEAM-E to three patients with recurrent GBM found no associated dose-limiting toxic effects. All participants demonstrated rapid radiographic tumor regression within days of administration, confirming CNS activity of the therapy. However, disease progression recurred within one to two months in two patients, indicating that responses were transient in the absence of sustained CAR T-cell persistence. These findings provide proof-of-principle for dual-antigen targeting in glioblastoma and highlight the need for future studies to enhance durability of response through optimized delivery, lymphodepletion, or repeated dosing [5].

Device-based therapies and tumor-disrupting modalities 

Tumor Treating Fields

Recently, several non-pharmacologic interventions designed to disrupt tumor growth through thermal, electromagnetic, or mechanical means have emerged. Novocure’s Optune device was previously presented in a progress report from 2016 [6], and updated evidence further supports the role of tumor-treating fields (TTFields) in GBM. TTFields are low-intensity, intermediate-frequency alternating electric fields that interfere with cellular processes critical for cancer cell viability and progression. Delivered regionally and non-invasively through a transducer array connected to a portable system, TTFields disrupt tumor cell mitosis and promote cell death while sparing non-dividing normal tissue [7].

The FDA first approved TTFields for recurrent GBM in 2011 (NovoTTF-100A System), and later in 2015 for newly diagnosed GBM in combination with TMZ [8, 9]. A meta-analysis of TTFields in newly diagnosed GBM confirmed improved overall survival (OS) across diverse populations [10]. Large post-marketing surveillance datasets, including analyses of over 11,000 patients [11] and more than 25,000 patients total [12], consistently demonstrated favorable tolerability, with no systemic toxicities and safety profiles consistent across age groups, sexes, and diagnoses. 

TTFields have demonstrated survival benefits in multiple randomized trials. The pivotal EF-14 Phase III study established that the addition of TTFields to maintenance TMZ therapy significantly improves both PFS (6.7 vs. 4.0 months, p<.001) and OS (20.9 vs. 16.0 months, p<.001) compared to TMZ alone, without additional systemic toxicity or adverse events [13, 14]. Post-hoc analyses showed that device adherence is a critical determinant of clinical benefit. Compliance of at least 50% improved PFS and OS compared with TMZ alone, highlighting the importance of patient education and support to maximize efficacy [15].

Oncomagnetic Therapy

Oncomagnetic therapy is another non-invasive modality being explored to disrupt tumor biology. Oscillating magnetic fields (OMFs) target resistant brain tumors using magnetic frequencies that attack complex I in the mitochondria of cancerous cells [16]. The subsequent accumulation of reactive oxygen species (ROS) initiates the caspase-mediated apoptotic pathway, leading to cellular destruction [16, 17]. Critically, the OMFs are selectively cytotoxic to cancer cells and do not affect normal cells [17]. The magnetic waves are delivered via three OMF generating components (oncoscillators) placed on a helmet worn by the patient for two hours at a time, two or three times a day - a key advantage over previous devices that must be worn at least 20 hours a day [16, 18].

Six end-stage GBM patients received oncomagnetic device therapy through a compassionate clinical trial under FDA-expanded access authorization. The cohort saw a 30-60% decrease in tumor volume prior to patient death. In the fall of 2022, the FDA granted expanded use access for the first non-GBM patient to receive oncomagnetic device therapy. A patient with chemotherapy- and radiation-resistant diffuse intrinsic pontine glioma (DIPG) with a three-month prognosis had minimal radiographic evidence of disease six months after oncomagnetic therapy [16]. Future directions include acquiring funding to further explore safety, efficacy, and organized clinical trials.

Photodynamic Therapy

Photodynamic therapy (PDT) is a light-based treatment that combines a photosensitizing agent with targeted light exposure to generate reactive oxygen species (ROS) that induce selective tumor cell death [19]. To date, numerous photosensitizers have been used in glioma studies, such as 5-aminolevulinic acid (5-ALA), boronated porphyrin, talaporfin, and temoporfin [20]. Among PDT agents, 5-aminolevulinic acid (5-ALA) is widely used for its dual diagnostic and therapeutic potential. It classically enables fluorescence-guided surgery through the accumulation of protoporphyrin IX (PpIX), enhancing intraoperative tumor visualization [19]. In fluorescence-guided surgery, excitation of PpIX by blue-violet light results primarily in photon emission for visualization, whereas PDT employs red light to excite PpIX into a triplet state that transfers energy to molecular oxygen, forming ROS [19].

Other agents, such as talaporfin sodium, a photosensitive radical-generating agent, have shown promising results. In a retrospective cohort of 100 patients, PDT with talaporfin sodium significantly reduced local tumor recurrence compared to standard treatment (56.4% vs. 83.9%, p = 0.0033), although distant recurrence and dissemination were significantly higher in the PDT group (48.7% vs. 16.1%, p = 0.0033). Notably, patients receiving PDT had longer median PFS (10.8 vs. 9.3 months, p = 0.016) and OS (24.6 vs. 17.6 months, p = 0.034) compared to controls [20]. While PDT has shown promise, particularly for recurrent disease, its efficacy remains limited by variability in PpIX accumulation, non-uniform light penetration, and inconsistent clinical outcomes [19, 20].

Sonodynamic Therapy

Sonodynamic therapy (SDT) represents an emerging, non-invasive approach for malignant brain tumors that circumvents the challenges of PDT. SDT also uses 5-ALA as a photosensitive agent; however, rather than visible light, SDT uses low-intensity focused ultrasound (LIFU) to activate PpIX through sonoluminescence and generate ROS [21]. Compared with PDT, SDT allows for deeper tissue penetration and broader field activation, allowing for the treatment of more diffuse disease presentations.

In the first-in-human study of the platform by Alpheus Medical, Inc. (Chanhassen, MN, USA; Phase 1/2 clinical trial, NCT05362409) in recurrent high-grade glioma, median OS reached 15.7 months, and a median PFS of 5.5 months, versus historical benchmarks of (6-8 and 1.8 months, respectively) [22]. Additionally, SonALAsence’s (Oakland, CA, USA) ongoing Phase 1/2 SDT-201 study in diffuse intrinsic pontine glioma (DIPG) has demonstrated encouraging early efficacy signals, with two patients showing at least 25% tumor volume reduction at eight weeks and two maintaining stable disease. Four patients exceeded the typical 9-to-11-month median survival and demonstrated maintained or improved performance status [23]. Neither treatment reported treatment-related deaths, duration-limited toxicities, or serious adverse events were reported. These findings highlight how 5-ALA may be impactful beyond fluorescence-guided surgery.

Drug delivery and localized therapy

Intra-Arterial Radioembolization

Intra-arterial radioembolization with Yttrium-90 (Y-90) microspheres, originally developed for hepatic malignancies, is being adapted for brain tumor therapy by delivering high-dose β-emitting microspheres into the tumor arterial supply to provide focal internal irradiation while largely sparing surrounding normal tissue [24]. Preclinical work in canine models demonstrated a 24-94% reduction in tumor volume at one-month post treatment without cortical atrophy or long-term neurological deficits, introducing intra-arterial delivery of Y-90 microspheres as both feasible and safe in cerebral circulation [25]. Building on this foundation, the FRONTIER Study (NCT05303467) was initiated as the first-in-human single-arm early-feasibility Phase I study to evaluate the safety and preliminary efficacy of Y-90 microspheres in patients with recurrent GBM. These localized strategies align with broader efforts to control glioma recurrence at the tumor margin.

Laser Interstitial Thermal Therapy and Thermosensitive Liposomes

To address tumors that are deep-seated, recurrent, or not amenable to open surgery, laser interstitial thermal therapy (LITT), guided by MR thermometry, is a less invasive alternative and allows for precise ablation of tumor tissue while sparing surrounding structures [26-30]. However, LITT is limited by relatively small ablation volumes, risk of thermal injury to adjacent eloquent structures, and postoperative edema that may cause neurological deficits [31]. Despite these limitations, the hyperthermia generated by LITT transiently disrupts the blood-brain barrier (BBB), creating a unique therapeutic window for enhanced local drug delivery [31, 32].

A promising strategy to harness the therapeutic potential of hyperthermia has recently emerged using low temperature-sensitive liposomes (LTSLs), a subclass of temperature-sensitive liposomes (TSLs) engineered to release their drug at mild hyperthermic temperatures (40-42°C). LTSL-Dox (ThermoDox®), developed by DNKO (https://www.dnko.net), encapsulates the chemotherapeutic agent doxorubicin, remaining stable under physiological conditions but rapidly releasing more than 80% of its contents when exposed to localized heating [33]. When combined with LITT, LTSLs provide a dual mechanism of action for the treatment of recurrent gliomas and allow for targeting of peripheral regions where gliomas most often recur [33, 34].

In early mouse models, all eleven mice implanted with human squamous cell carcinoma (SCC) xenografts showed complete regression when treated with LTSL-Dox and tumor heating to 42 °C for one hour [35]. While such results highlight the potential of hyperthermia-triggered drug release, significant translational work, including validation in human clinical trials, optimization of dosing parameters, and evaluation of safety and efficacy in patients, is needed before widespread application.

Cytotoxic agents

*Novel Taxane Derivatives* 

Taxanes, a widely used class of chemotherapeutics first approved in 1984, inhibit tumor growth through various mechanisms, including microtubule stabilization, cell cycle arrest, apoptosis induction, and angiogenesis inhibition [36]. They promote mitochondrial ROS production and p53 activation, which upregulate cell cycle inhibitor p21Cip1 and downregulate anti-apoptotic BCL2 proteins [37, 38]. Taxanes also suppress vascular proliferation by reducing pro-angiogenic proteins VEGF and Ang-1 while increasing the release of the anti-angiogenic protein TSP-1 [39].

Taxanes are effective in breast, ovarian, lung, prostate, and head and neck cancers, as well as GBM cell lines in vitro [36, 40]. However, they have not been validated for GBM in vivo due to poor BBB penetration and vulnerability to multidrug resistance (MDR) efflux pumps. Taxanes’ clinical durability is further limited by chemoresistance [37, 41]. TPI 287 (abeotaxane), a novel taxane derivative, addresses these limitations by combining high lipophilicity with non-substrate activity for MDR efflux pumps, enabling BBB permeability and efficacy in taxane-resistant tumor cells [42].

TPI-287 has been studied in over 350 clinical trial patients as both a monotherapy and in combination with bevacizumab, demonstrating minimal toxicity. It is currently being tested in a multi-center phase I/II clinical trial in combination with bevacizumab for recurrent GBM. Phase I results demonstrate safety and tolerability at doses up to 220 mg/m2, with improved PFS and OS compared with historical controls of bevacizumab alone [41]. For these reasons, TPI-287 may also have potential applications in CNS metastases, particularly from taxane-sensitive primary tumors such as breast cancer [42].

Imaging biomarkers

Fractional Tumor Burden Mapping

Distinguishing true tumor progression from treatment-related changes (e.g., pseudoprogression and radiation necrosis) remains a major challenge [43]. Conventional MRI lacks specificity in this context, often leading to diagnostic uncertainty and delays in care [44]. Quantitative imaging biomarkers have emerged to improve response assessment and guide therapy [45, 46].

Imaging Biometrics (Elm Grove, WI, USA; a subsidiary of IQ-AI, Inc.) has developed fractional tumor burden (FTB) mapping, an MRI-based biomarker derived from quantitative perfusion imaging. Using automated analysis of relative cerebral blood volume (rCBV), FTB mapping spatially differentiates regions within enhancing lesions. Higher perfusion in the tumor mass or residual can therefore be differentiated from low perfusion regions consistent with treatment effects [47, 48]. The resulting voxel-wise maps overlay conventional MRI, providing a clear visualization of tumor burden versus treatment effect. The FDA-approved platform has been integrated into clinical picture archiving and communication system/radiology information system (PACS/RIS) workflow systems, enabling routine clinical use without additional infrastructure [49].

Multiple studies have demonstrated that FTB mapping improves diagnostic accuracy over standard MRI in differentiating true progression from pseudoprogression, particularly in high-grade gliomas [50, 51]. Evidence supports its application in treatment monitoring, surgical planning, and radiotherapy assessment. FDA clearance for FTB reflects both technical validation and reproducibility across scanners and institutions [48, 49]. Future directions include multicenter validation of prognostic value, correlation with survival outcomes, and integration with multiparametric MRI, radiomics, and AI-based predictive models [52].

Precision medicine

Ex Vivo Prediction of Drug Response

Therapeutic decision-making in gliomas traditionally relies on histopathology and molecular profiling. However, both approaches are limited in their ability to identify functional drug response prospectively [53, 54]. Functional precision medicine platforms that directly evaluate patient-derived tumor tissue are emerging to address this shortfall. Kiyatec (Greenville, SC, USA) has developed a three-dimensional (3D) ex vivo culture system to predict chemotherapy efficacy by modeling the tumor microenvironment [55].

Kiyatec’s clinical assay, 3D PredictTM Glioma, cultures freshly resected or biopsied tumor tissue in a tumor-mimicking 3D microenvironment to test a panel of 12 chemotherapeutic and targeted agents, including TMZ, lomustine, irinotecan, and target inhibitors (e.g., osimertinib, dabrafenib, everolimus) [55]. The assay measures tumor cell viability and therapeutic sensitivity to complement molecular diagnostics rather than replace them [55].

The platform first demonstrated clinical predictive validity in ovarian cancer studies [55]. In gliomas, the 3D-PREDICT study (NCT03561207) has reported encouraging interim data showing concordance between ex vivo drug response and clinical outcomes. Recent analyses in high-grade glioma cohorts indicate that pre-treatment functional predictions correlate with PFS and OS, supporting the assay’s potential clinical utility [55].

Remaining challenges include tissue procurement logistics, patient- and hospital-facing costs, and the need for interventional studies demonstrating direct impact on patient outcomes [55]. Despite these challenges, Kiyatec exemplifies how functional precision medicine can complement molecular profiling in neuro-oncology [55].

In Vivo Precision Imaging and Response Assessment 

Building on advances in functional precision medicine, neuroimaging innovations are also changing how clinicians assess and monitor glioma biology in vivo. Among these, dedicated brain PET-CT systems represent an important step forward. Conventional whole-body PET/CT has long been applied in oncology but offers limited performance for brain tumors due to modest spatial resolution, relatively high noise, and patient positioning constraints [56]. For gliomas and other CNS malignancies, tumor delineation and early detection are critical for guiding surgery, radiotherapy planning, and monitoring therapeutic response [56]. Dedicated brain PET systems aim to overcome these barriers by providing higher sensitivity, improved resolution, and enhanced patient tolerability [56].

Oncovision (Boston, MA, USA) has introduced CareMiBrain, a PET system designed specifically for brain imaging [56]. Unlike whole-body scanners, the device uses a compact ring geometry optimized for the head, providing improved resolution and sensitivity [56]. Patients are scanned in a seated position, aiming to reduce motion artifacts and improve comfort. The system achieves diagnostic information and image quality comparable to conventional PET/CT at a lower tracer dose, potentially allowing for safer repeated imaging during therapy courses [57].

Performance evaluation has demonstrated that CareMiBrain exceeds many whole-body PET systems in spatial resolution, sensitivity, and lesion detectability [56]. Early clinical adoption has highlighted utility in distinguishing recurrence from post-treatment change and assessing treatment response where MRI findings are inconclusive, such as differentiating tumor progression from pseudo-progression or radiation necrosis [58]. Ongoing challenges include broad device deployment, reimbursement, and demonstration of outcome-altering benefits in prospective neuro-oncology trials. Integration with advanced MRI, novel tracers, and AI-based image analysis may further enhance its role. Continued multicenter studies will be essential to determine the technology’s impact on patient management and survival outcomes.

Supportive platforms

Connectomics and Personalized Neurosurgery

Beyond LITT’s use in enhancing drug delivery, it has also been employed alongside connectomic mapping techniques, which are increasingly integrated into neurosurgical practice to optimize both oncologic control and functional preservation. 

Personalized connectome imaging integrates resting-state functional magnetic resonance imaging (fMRI) and diffusion tensor imaging (DTI) with individualized brain network atlases to delineate structural and functional connectivity unique to each patient. This approach enables tailored risk assessment and surgical planning by identifying critical white-matter tracts and functional hubs involved in language, motor, and memory networks [59]. In LITT, connectomic mapping can guide trajectory planning and ablation volume selection to minimize disruption of key neural circuits [59]. Evidence indicates that connectomic features such as the strength of connectivity can retrospectively predict seizure outcomes following LITT for epilepsy, while comparative studies suggest superior network preservation with LITT compared to open craniotomy for tumor resection [60]. Incorporating connectomic analysis into LITT workflows represents a shift toward individualized, network-preserving neurosurgery aimed at optimizing both oncologic and functional outcomes.

## Conclusions

Since its establishment in 2012, the Brain Tumor Biotech Summit has become a leading forum at the intersection of translational science, biotechnology, and investment, driving innovation forward in neuro-oncology. The 2025 meeting reflects a shift towards a more integrated ecosystem where academic discovery and industry development converge to accelerate care for patients who need it most. Moving forward, the summit is poised to expand its role as an incubator, fostering rapid advancement of precision therapeutics, biologics, and technologies that redefine standards of care. Sustained collaboration among researchers, clinicians, and biotechnology innovators will be vital to transforming early-stage concepts into viable treatments that meaningfully improve outcomes for patients with brain tumors.

## References

[1] Pouyan A, Ghorbanlo M, Eslami M (2025). Glioblastoma multiforme: insights into pathogenesis, key signaling pathways, and therapeutic strategies. Mol Cancer.

[2] Stupp R, Mason WP, van den Bent MJ (2005). Radiotherapy plus concomitant and adjuvant temozolomide for glioblastoma. N Engl J Med.

[3] Andrews DW, Judy KD, Scott CB (2021). Phase Ib clinical trial of IGV-001 for patients with newly diagnosed glioblastoma. Clin Cancer Res.

[4] Lee IY, Hanft S, Schulder M (2024). Autologous cell immunotherapy (IGV-001) with IGF-1R antisense oligonucleotide in newly diagnosed glioblastoma patients. Future Oncol.

[5] Choi BD, Gerstner ER, Frigault MJ (2024). Intraventricular CARv3-TEAM-E T cells in recurrent glioblastoma. N Engl J Med.

[6] Chakraborty S, Bodhinayake I, Chiluwal A, Langer DJ, Ruggieri R, Symons M, Boockvar JA (2016). Neuro-oncology biotech industry progress report. J Neurooncol.

[7] Karanam NK, Story MD (2021). An overview of potential novel mechanisms of action underlying tumor treating fields-induced cancer cell death and their clinical implications. Int J Radiat Biol.

[8] Fonkem E, Wong ET (2012). NovoTTF-100A: a new treatment modality for recurrent glioblastoma. Expert Rev Neurother.

[9] Mehta M, Wen P, Nishikawa R, Reardon D, Peters K (2017). Critical review of the addition of Tumor Treating Fields (TTFields) to the existing standard of care for newly diagnosed glioblastoma patients. Crit Rev Oncol Hematol.

[10] Ballo MT, Conlon P, Lavy-Shahaf G, Kinzel A, Vymazal J, Rulseh AM (2023). Association of Tumor Treating Fields (TTFields) therapy with survival in newly diagnosed glioblastoma: a systematic review and meta-analysis. J Neurooncol.

[11] Shi W, Blumenthal DT, Oberheim Bush NA (2020). Global post-marketing safety surveillance of Tumor Treating Fields (TTFields) in patients with high-grade glioma in clinical practice. J Neurooncol.

[12] Mrugala MM, Shi W, Iwomoto F, Lukas RV, Palmer JD, Suh JH, Glas M (2024). Global post‑marketing safety surveillance of tumor treating fields (TTFields) therapy in over 25,000 patients with CNS malignancies treated between 2011-2022. J Neurooncol.

[13] Taphoorn MJ, Dirven L, Kanner AA (2018). Influence of treatment with tumor-treating fields on health-related quality of life of patients with newly diagnosed glioblastoma: a secondary analysis of a randomized clinical trial. JAMA Oncol.

[14] Stupp R, Taillibert S, Kanner A (2017). Effect of tumor-treating fields plus maintenance temozolomide vs maintenance temozolomide alone on survival in patients with glioblastoma: a randomized clinical trial. JAMA.

[15] Toms SA, Kim CY, Nicholas G, Ram Z (2019). Increased compliance with tumor treating fields therapy is prognostic for improved survival in the treatment of glioblastoma: a subgroup analysis of the EF-14 phase III trial. J Neurooncol.

[16] (2025). Oncomagnetic device a promising non-invasive weapon against deadly brain cancer cells. https://www.houstonmethodist.org/leading-medicine-blog/articles/2023/mar/oncomagnetic-device-a-promising-non-invasive-weapon-against-deadly-brain-cancer-cells/.

[17] Hambarde S, Manalo JM, Baskin DS, Sharpe MA, Helekar SA (2023). Spinning magnetic field patterns that cause oncolysis by oxidative stress in glioma cells. Sci Rep.

[18] Baskin DS, Sharpe MA, Nguyen L, Helekar SA (2021). Case Report: End-Stage Recurrent Glioblastoma Treated With a New Noninvasive Non-Contact Oncomagnetic Device. Front Oncol.

[19] Cramer SW, Chen CC (2019). Photodynamic therapy for the treatment of glioblastoma. Front Surg.

[20] Fujimoto Y, Fujita Y, Tanaka K (2025). Clinical benefits of photodynamic therapy using talaporfin sodium in patients with isocitrate dehydrogenase -wildtype diagnosed glioblastoma: a retrospective study of 100 cases. Neurosurgery.

[21] Cressey P, Abd Shukor SB, Thanou M (2025). Sonodynamic therapy: transforming sound into light for hard-to-treat tumours. Adv Drug Deliv Rev.

[22] (2025). Alpheus Medical announces positive Phase 1/2 trial results for the treatment of recurrent high-grade gliomas. https://alpheusmedical.com/news/alpheus-medical-announces-positive-phase-1-2-trial-results-for-the-treatment-of-recurrent-high-grade-gliomas/.

[23] (2025). SonALAsense presents preliminary data from clinical study in patients with deadly pediatric brain tumor. https://www.sonalasense.com/news/sonalasense-presents-preliminary-data-from-clinical-study-in-patients-with-deadly-pediatric-brain-tumor-.

[24] Murthy R, Kamat P, Nuñez R, Salem R (2008). Radioembolization of yttrium-90 microspheres for hepatic malignancy. Semin Intervent Radiol.

[25] Pasciak AS, Manupipatpong S, Hui FK (2020). Yttrium-90 radioembolization as a possible new treatment for brain cancer: proof of concept and safety analysis in a canine model. EJNMMI Res.

[26] Carpentier A, Chauvet D, Reina V (2012). MR-guided laser-induced thermal therapy (LITT) for recurrent glioblastomas. Lasers Surg Med.

[27] Seaton MP, Schmidt JC, Brown NJ, Sahyouni R, Khalessi AA, Ben-Haim S, Gonda DD (2025). Contemporary applications of laser interstitial thermal therapy: a comprehensive systematic review. World Neurosurg.

[28] Nielsen SH, Rasmussen R (2024). MR-guided laser interstitial thermal therapy in the treatment of brain tumors and epilepsy. Acta Neurochir (Wien).

[29] Schupper AJ, Chanenchuk T, Racanelli A, Price G, Hadjipanayis CG (2022). Laser hyperthermia: past, present, and future. Neuro Oncol.

[30] Bozinov O, Yang Y, Oertel MF, Neidert MC, Nakaji P (2020). Laser interstitial thermal therapy in gliomas. Cancer Lett.

[31] Rifi Z, Harary M, Walshaw PD (2024). Functional magnetic resonance imaging (fMRI) as adjunct for planning laser interstitial thermal therapy (LITT) near eloquent structures. Acta Neurochir (Wien).

[32] Leuthardt EC, Duan C, Kim MJ (2016). Hyperthermic laser ablation of recurrent glioblastoma leads to temporary disruption of the peritumoral blood brain barrier. PLoS One.

[33] (2025). Our approach combining LTSL-Dox + LITT. https://www.dnko.net/our-scientific-approach.

[34] Regenold M, Bannigan P, Evans JC, Waspe A, Temple MJ, Allen C (2022). Turning down the heat: The case for mild hyperthermia and thermosensitive liposomes. Nanomedicine.

[35] Needham D, Anyarambhatla G, Kong G, Dewhirst MW (2000). A new temperature-sensitive liposome for use with mild hyperthermia: characterization and testing in a human tumor xenograft model. Cancer Res.

[36] Zumbar CT, Usubalieva A, King PD (2018). The CNS penetrating taxane TPI 287 and the AURKA inhibitor alisertib induce synergistic apoptosis in glioblastoma cells. J Neurooncol.

[37] Mosca L, Ilari A, Fazi F, Assaraf YG, Colotti G (2021). Taxanes in cancer treatment: activity, chemoresistance and its overcoming. Drug Resist Updat.

[38] Fitzpatrick JM, de Wit R (2014). Taxane mechanisms of action: potential implications for treatment sequencing in metastatic castration-resistant prostate cancer. Eur Urol.

[39] Bocci G, Di Paolo A, Danesi R (2013). The pharmacological bases of the antiangiogenic activity of paclitaxel. Angiogenesis.

[40] Ahmed M, Semreen AM, Giddey AD (2023). Proteomic and metabolomic signatures of U87 glioblastoma cells treated with cisplatin and/or paclitaxel. Ann Med.

[41] Goldlust SA, Nabors LB, Hsu S (2024). Phase 1 trial of TPI 287, a microtubule stabilizing agent, in combination with bevacizumab in adults with recurrent glioblastoma. Neurooncol Adv.

[42] Fitzgerald DP, Emerson DL, Qian Y (2012). TPI-287, a new taxane family member, reduces the brain metastatic colonization of breast cancer cells. Mol Cancer Ther.

[43] Verma N, Cowperthwaite MC, Burnett MG, Markey MK (2013). Differentiating tumor recurrence from treatment necrosis: a review of neuro-oncologic imaging strategies. Neuro Oncol.

[44] Galldiks N, Kaufmann TJ, Vollmuth P (2024). Challenges, limitations, and pitfalls of PET and advanced MRI in patients with brain tumors: A report of the PET/RANO group. Neuro Oncol.

[45] Young JS, Al-Adli N, Scotford K, Cha S, Berger MS (2023). Pseudoprogression versus true progression in glioblastoma: what neurosurgeons need to know. J Neurosurg.

[46] van Dijken BR, van Laar PJ, Smits M, Dankbaar JW, Enting RH, van der Hoorn A (2019). Perfusion MRI in treatment evaluation of glioblastomas: Clinical relevance of current and future techniques. J Magn Reson Imaging.

[47] Amidon RF, Santos-Pinheiro F, Straza M (2022). Case report: Fractional brain tumor burden magnetic resonance mapping to assess response to pulsed low-dose-rate radiotherapy in newly-diagnosed glioblastoma. Front Oncol.

[48] (2025). FTB maps. https://imagingbiometrics.com/our-solutions/ftb-maps/..

[49] Iv M, Liu X, Lavezo J (2019). Perfusion MRI-based fractional tumor burden differentiates between tumor and treatment effect in recurrent glioblastomas and informs clinical decision-making. AJNR Am J Neuroradiol.

[50] Connelly JM, Prah MA, Santos-Pinheiro F, Mueller W, Cochran E, Schmainda KM (2021). Magnetic resonance imaging mapping of brain tumor burden: clinical implications for neurosurgical management: case report. Neurosurg Open.

[51] Herings SD, van der Wijk MW, von Beckerath V, Fasen BA, Meijer FJ, van der Kolk AG, Henssen DJ (2024). Fractional tumor burden maps increase the confidence of reading brain MR perfusion. Eur J Radiol.

[52] Pang Y, Wang H, Li H (2021). Medical imaging biomarker discovery and integration towards AI-based personalized radiotherapy. Front Oncol.

[53] Shuford S, Lipinski L, Abad A (2021). Prospective prediction of clinical drug response in high-grade gliomas using an ex vivo 3D cell culture assay. Neurooncol Adv.

[54] Ledford A, Rodriguez A, Lipinski L (2024). Functional prediction of response to therapy prior to therapeutic intervention is associated with improved survival in patients with high-grade glioma. Sci Rep.

[55] (2025). 3D-PREDICT platform. https://kiyatec.com/healthcare-professionals/3d-predict-platform/.

[56] Moliner L, Rodríguez-Alvarez MJ, Catret JV, González A, Ilisie V, Benlloch JM (2019). NEMA performance evaluation of CareMiBrain dedicated brain PET and comparison with the whole-body and dedicated brain PET systems. Sci Rep.

[57] Gandia-Ferrero MT, Torres-Espallardo I, Martínez-Sanchis B (2023). Objective image quality comparison between brain-dedicated PET and PET/CT scanners. J Med Syst.

[58] (2025). CareMiBrain. https://oncovision.com/caremibrain/.

[59] Samuel N, Vetkas A, Pancholi A (2021). A network-based approach to glioma surgery: insights from functional neurosurgery. Cancers (Basel).

[60] Yang PH, Hacker CD, Patel B, Daniel AG, Leuthardt EC (2021). Resting-state functional magnetic resonance imaging networks as a quantitative metric for impact of neurosurgical interventions. Front Neurosci.

